# Mesenchymal stem cells alleviate rat diabetic nephropathy by suppressing CD103^+^ DCs‐mediated CD8^+^ T cell responses

**DOI:** 10.1111/jcmm.15250

**Published:** 2020-04-13

**Authors:** Fuping Zhang, Chengshi Wang, Xin Wen, Yang Chen, Ruiwen Mao, Danli Cui, Lan Li, Jingping Liu, Younan Chen, Jingqiu Cheng, Yanrong Lu

**Affiliations:** ^1^ Key Laboratory of Transplant Engineering and Immunology Regenerative Medicine Research Center West China Hospital Sichuan University Chengdu China

**Keywords:** CD103^+^ dendritic cells, diabetic nephropathy, immunosuppression, kidney injury, mesenchymal stem cells

## Abstract

Diabetic nephropathy (DN) as a kind of serious microvascular complication of Diabetes Mellitus (DM) usually causes the end‐stage of renal disease (ESRD). Studies have demonstrated that CD103^+^ dendritic cells (DCs) exhibited a renal pathogenic effect in murine chronic kidney disease (CKD). Mesenchymal stem cells (MSCs) can alleviate DN and suppress the DCs maturation. To explore the role of CD103^+^ DCs and the potential mechanisms underlying MSCs‐mediated protective effects in DN, we used bone marrow MSCs (BM‐MSCs) to treat DN rats. MSCs transplantation considerably recovered kidney function and diminished renal injury, fibrosis and the population of renal CD103^+^ DCs in DN rat. The MSCs‐treated DN rats had decreased mRNA expression levels of interleukin (*IL*)*1β*, *IL6*, tumour necrosis factor *alpha* (*TNF‐α*), monocyte chemotactic protein 1 (*MCP‐1*) and reduced CD8 T cell infiltration in the kidney. MSCs significantly down‐regulated the genes expression of transcription factors (Basic leucine zipper transcriptional factor ATF‐like 3, *Batf3* and DNA‐binding protein inhibitor ID‐2, *Id2*) and FMS‐like tyrosine kinase‐3 (*Flt3*) which are necessary for CD103^+^ DCs development. The protective effect of MSCs may be partly related to their immunosuppression of CD8^+^ T cell proliferation and activation mediated by CD103^+^ DCs in the kidney of DN rats.

## 1 INTRODUCTION

Diabetic nephropathy as a kind of the most serious microvascular complications of DM is one of the main causes of ESRD.^1^ Studies have shown that approximately one third of individuals with DM ultimately develop DN.^2^ Current treatment often cannot prevent the progression of DN, and the consistently high risk for most DN patients highlights the urgent need for more new therapies.^3^

Both adaptive and innate immune system have made a key contribution to the progression of DN. Researches have demonstrated that the development of DN was related to the infiltration of T cells (including CD8^+^ T cells) in the kidney^4^, ^5^, ^6^, ^7^ and activation of T cells in the circulation.^8^ DCs are critical for both adaptive and innate immune responses.^9^ Actually, renal DCs create a complicated network in tubule‐interstitium, where they continually engulf antigens and present them to T lymphocytes.^10^, ^11^ In kidney diseases, including DN, DCs prefer to migrate to the injury sites and express more chemokines, cytokines and co‐stimulatory molecules, acting on renal cells and innate immune cells.^12^ Multiple studies have shown that DCs are pathogenic in different kidney diseases, suggesting that DCs are important for the initiation and progression of renal disease.^13^, ^14^, ^15^, ^16^ To distinct from renal macrophages, renal DCs are defined as MHC‐II^+^ CD11c^+^ F4/80^−^ cell subsets in recent research.^17^ And renal DCs can be further divided into two subsets depending on the expression of CD103. Most importantly, CD103^+^ DCs accelerate renal injury via activating CD8 T cells in adriamycin nephropathy (AN).^13^ And suppression of CD103^+^ DCs with Flt3 inhibitor AC220 significantly alleviated renal injury in AN mice, which suggested that CD103^+^ DCs could be targeted as an effective treatment of CKD.^18^ However, more experiments are needed to verify the effect of CD103^+^ DCs in DN.

Mesenchymal stem cells are adult stem cells that possess many properties that including immunomodulation, secretion of soluble factors and anti‐apoptosis. These properties make MSCs treatment as a good strategy to intervene renal injury in DN. Multiple studies recently showed that MSCs had inhibitory effects on DCs.^19^, ^20^, ^21^, ^22^, ^23^ MSCs suppress the migration and the antigen (Ag)‐presenting function of DCs, serving as an immunoregulator. Previous studies demonstrated that early intervention with MSCs prevents renal injury in DN rats via improving the hyperglycaemia‐induced endothelial injury and inflammatory microenvironment.^24^, ^25^

In this study, we analysed a possibly immunomodulating mechanism by which MSCs could reduce the population and maturation of CD103^+^ DCs, alleviate CD103^+^ DCs‐mediated CD8 T cell responses, ameliorate local inflammation and exhibit a protective effect in the diseased kidney of DN rat.

## 2 MATERIAL AND METHODS

### 2.1 Rats and induction of DN

Six‐week‐old male Sprague Dawley (SD) rats (200 g bodyweight) were purchased from Chengdu Dashuo Biological Technology Co., Ltd. (Chengdu, China). Animals were housed in pairs of two in cages with controlled temperature (20‐22˚C), humidity (40%‐60%) and 12 hours cycles of light and darkness, and fed with standard chow and tap water ad libitum during one‐week acclimation period. Diabetes (fasting blood glucose levels >11.1 mmol/L on two consecutive days) was induced by intraperitoneally injected with 55 mg/kg bodyweight of streptozotocin (STZ, Sigma) once. Diabetic rats were housed for six weeks to induce renal injury. All rats were fed with regular diet (10% fat, 20% protein and 70% carbohydrate).

All animal studies and experimental protocols were approved and reviewed by the Animal Ethics Committee of the Sichuan University, which are consistent with the National Institutes of Health Guide for the Care and Use of Laboratory Animals.

### 2.2 Experimental design and transplantation of MSCs

**Figure 1:**
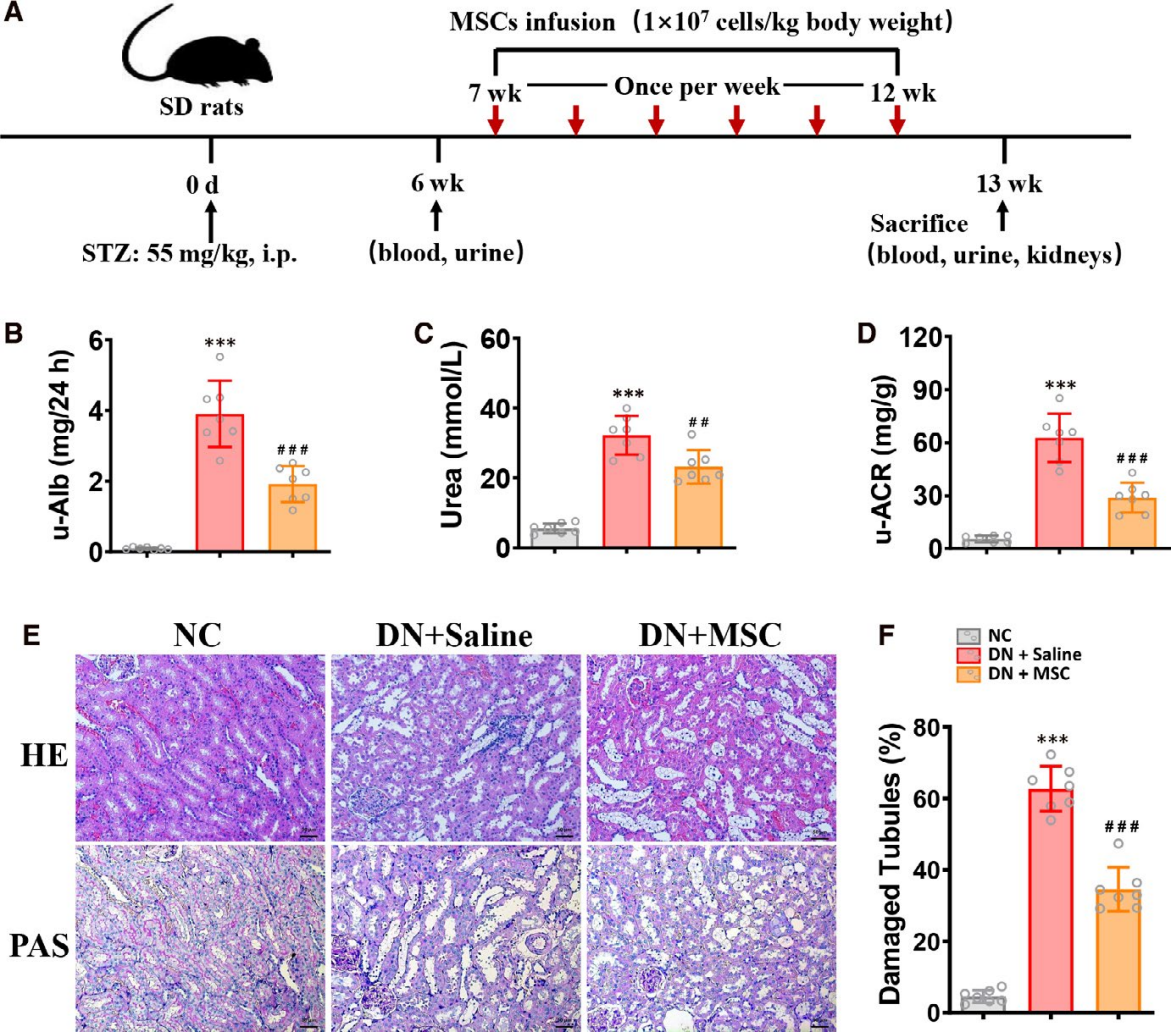
MSCs transplantation protected against renal injury in DN rats. A, DN rats were treated with MSCs weekly for 6 consecutive weeks after being induced renal injury with STZ injected interperitoneally for prier 6 weeks. Rats were killed after 12 weeks. B‐D, levels of u‐Alb, blood Urea and u–ACR were assessed in NC, DN + Saline and DN + MSC rats after 12 weeks. Data shown are the mean ± SD (n = 7 per group). ***P < .001 vs NC group, ##*P* < .01, ###*P* < .001 vs DN + Saline group. E, Representative HE and PAS sections of kidney from normal and DN rats treated with Saline or MSCs after 12 weeks. Bar = 50 µm. F, Kidney injury (damaged tubules) was assessed quantitatively. Data shown are the mean ± SD (n = 7 hpf per group, hpf, high‐power field). ***P < .001 vs NC group, ###*P* < .001 vs DN + Saline group

In addition to normal control group (NC, n = 7, healthy rats without treatment); diabetic rats were randomly assigned into DN group (DN + Saline, n = 7, diabetic rats receiving injection of 0.5 mL 0.9% saline); BM‐MSCs treatment group (DN + MSC, n = 7, diabetic rats receiving injection of BM‐MSCs). Six weeks after the induction of diabetes, 1 × 10^7^ cells/kg bodyweight MSCs were suspended in 1 mL saline and transplanted to rats via tail vein injection weekly for six weeks. Meanwhile, DN group rats were infused with 1mL saline. After 12 weeks, the rats were killed and their blood, urine and kidney were collected for further detection (Figure 1A).

### 2.3 Isolation, culture and FACS analysis of BM‐MSCs

The rat BM‐MSCs were isolated and cultured as described previously.^25^ Briefly, male SD rats (80‐100 g bodyweight) were killed with an overdose of choral hydrate. The femurs and tibias were isolated and rinsed with phosphate‐buffered saline (PBS). After muscles were trimmed, BM mononuclear cells were flushed from bone cavities with PBS. The harvested cells were centrifuged, washed, re‐suspended in low‐glucose Dulbecco's minimal essential medium (LG‐DMEM, Gibco) containing 10% foetal bovine serum (FBS) (Gibco), 100 U/mL penicillin, and 100 mg/mL streptomycin and then seeded in T25 flasks and incubated at 37°C in 5% humidified CO_2_ for 72 hours without checking. Then, the non‐adherent cells were removed and fresh medium was added. The medium was changed every other day. At 80% confluence, cells were treated with 0.25% trypsin (Gibco) and passaged at a ratio of 1:3. MSCs between passages three and five were used for transplantation.

FACS analysis of rat BM‐MSCs was performed at the fifth passage. Cells were rinsed with PBS, detached from the T25 flasks using 0.25% trypsin (Gibco), centrifuged at 200 × *g* for 5 minutes, rinsed and re‐suspended in PBS. Re‐suspended cells were incubated with 5 μL of fluorescein isothiocyanate‐labelled anti‐rat CD29 (BD), CD90 (BD), CD44 (BD) and phycoerythrin (PE)‐conjugated anti‐rat CD34 (BD), CD45 (BD) in the dark at 4°C for 30 miutes and washed. The labelled cells were analysed on a FACS Aria machine (BD Biosciences).

### 2.4 Flow cytometry and cell sorting

The single‐cell suspensions preparation and flow cytometry analysis was administrated as described previously.^13^ Briefly, kidneys were cut into 1‐2 mm^3^ pieces before placed in DMEM containing 100 mg/mL deoxyribonuclease (DNase) I (Roche) and 1 mg/mL collagenase IV (Sigma Aldrich) for 40 minutes at 37°C with intermittent agitation. The digested cell suspension was then passed through a 40‐μm cell strainer and washed with PBS twice.

For fluorescence‐activated cell sorting (FACS) analysis of kidney samples, single‐cell suspensions were incubated with bovine serum albumin (BSA) to block non‐specific binding and antibodies to CD45 (BD), MHC‐II (Novus), CD11c (Abcam), CD68 (Novus), CD11b (Novus) and CD103 (BD), as well as antibodies to natural killer (NK) cell, T cell and B cell lineages (lin): CD3 (Biolegend), T cell receptor (TCR)‐β (Biolegend), TCR‐γδ (Biolegend), CD19 (Santa) and CD49b (BD). When FACS sorting was performed on the digested kidney single‐cell suspension, cells were pregated on hematopoietic cells using anti‐CD45 antibody. Then, lineages (CD3/ CD19/CD49b/ TCR‐β/ TCR‐γδ) were used to exclude NK cells and lymphocytes, and 4’,6‐diamidino‐2‐phenylindole (DAPI) was used to exclude dead cells. Then after gated renal mononuclear phagocytes (rMPs) as lin^−^ MHCII^+^ cell subsets, Renal CD68^−^ CD11c^+^ (rMP1), CD68^+^ CD11c^+^ (rMP2), CD103^+^ CD11b^−^ (rMP3), CD103^−^ CD11b^+^ (rMP4) cell subsets and splenic CD8^+^ T cells were analysed or sorted. The sorted cells were then used for further analyzations.

Other antibodies used in another study include CD86 (BD), CD80 (Biolegend) and granzyme B (Abcam), as well as corresponding isotype controls. Cells were analysed on a FACS Aria machine (BD Biosciences).

### 2.5 Histological examination

Histological examination was performed as previously described.^26^ The fixed renal tissues were embedded in paraffin and made to 5 μm sections. Renal sections were deparaffinized in xylene and rehydrated in graded ethanol, and then stained with haematoxylin‐eosin (HE), Masson's trichrome (Masson) and periodic acid–Schiff (PAS). For immunohistochemical (IHC) staining, sections were blocked with 1% BSA, and incubated with diluted primary antibodies including rabbit anti‐Alpha‐smooth muscle actin (α‐SMA, Abcam, USA), then incubated with horseradish peroxidase (HRP)‐conjugated secondary antibody (DAKO, USA), and finally stained with 3,3’‐diaminobenzidine (DAB) substrate and haematoxylin. Immunofluorescence (IF) was performed with mouse anti‐rat CD8 (Abcam), mouse anti‐rat CD11c (Abcam) or/and rabbit anti‐rat CD103 (Abcam). The images of stained sections were acquired by microscope (Carl Zeiss, Germany), and quantitative analysis of damaged tubules (%) and positive cells (number per high‐power fields, hpf) in images was done by using ImageJ software (NIH, USA).

### 2.6 Biochemical measurement

Clinical biochemistry analysis of the urine and serum samples was performed on an Automatic Biochemistry Analyzer (Cobas Integra 400 plus, Roche) by commercial kits with the following parameters: creatinine (CREA), blood urea, blood urea nitrogen (BUN), urinary albumin to creatinine ratio (u‐ACR), triglyceride (TG), cholesterol (TC), low‐density lipoprotein cholesterol (LDL‐C) and high‐density lipoprotein cholesterol (HDL‐C).

### 2.7 **Preparation of bone marrow MSCs conditioned media (MSC**‐**CM)**

MSCs between passages of 3‐4 were used to prepare MSC‐CM as previously described.^27^ After incubation for 24 hours, the cell culture medium was collected and centrifuged at 1000 *× g* for 8 min at 4°C. Then, the supernatant was used as MSC‐CM.

### 2.8 Generation of rat BM‐derived DCs and Coculture assay

BM‐derived DCs were isolated and induced differentiation as previously described.^28^ BM mononuclear cells were separated and cultured with 20 ng/mL recombinant rat granulocyte‐macrophage colony‐stimulating factor (GM‐CSF; Biovision, USA) and 20 ng/mL recombinant rat interleukin 4 (IL4; Biovision, USA) for 5 days to induce immature dendritic cells (iDCs), which were assessed by flow cytometry. iDCs were induced at day 5 with 200 ng/mL TNF‐α (PEPROTECH, MU, USA) stimulation for another 2 days to became mature dendritic cells (mDCs). Flow cytometry analysis was performed to evaluate the DCs maturation with CD11c (Abcam), CD68 (Novus), CD103 (BD), CD11b (Novus), CD86 (BD) and CD80 (Biolegend).

CD103^+^ DCs sorted from mDCs were cultured with or without MSC‐CM (10:1) for 48 h, and the expression of surface markers (including CD80 and CD86) on CD103^+^ DCs was analysed.

### 2.9 Proliferation assay

Splenic lymphocyte cells were isolated by pushing rat spleens through 70 μm cell strainers to prepare single‐cell suspensions. In proliferation assays, CD103^+^ DCs (1 × 10^5^) isolated from mDCs were incubated with or without MSC‐CM for 48 hours and then cocultured with carboxyl fluorescent succinimidyl ester (CFSE)‐labelled splenic lymphocyte cells (1 × 10^6^) for 3 days. CD8^+^ T Cell proliferation was examined with CFSE (Invitrogen) and peridinin chlorophyll a protein (PerCP)‐conjugated anti‐rat CD8 antibody (Biolegend) by flow cytometry.

### 2.10 Quantitative real‐time PCR (qPCR)

Total RNA was extracted from renal cortical tissue (isolated CD103^+^ DCs or CD8 T cells in another study) by using Trizol reagent (Invitrogen) according to the manufacturer's instructions and then reverse transcribed into cDNA using Transcriptor First Strand cDNA synthesis kit (Roche, Indianapolis, IN, USA). qPCR was performed in triplicates using the iQ SYBR‐Green Super mix and specific primers (Shenggong Biotechnology Shanghai, China) with a Bio‐Rad CFX96TM Real‐time PCR Detection System. The sequences of primers were all listed in Table S1.

### 2.11 Statistical analyses

All data are presented from at least three independent experiments. Statistical tests included unpaired, two‐tailed Student's *t* test using Welch's correction for unequal variances and one‐way analysis of ANOVA with Tukey's multiple comparisons test. Statistical analyses were performed using Prism software (version 8; GraphPad). Results are presented as the mean ± SD *P* < .05 was considered statistically significant.

## 3 RESULTS

### 3.1 MSCs transplantation Protected Against Renal Injury in DN rats

We have demonstrated previously that early treatment with MSCs prevented renal injury by improving the inflammatory microenvironment in diabetic rats.^24^ Here, we investigated the role of MSCs in injured kidneys using the DN model. Flow cytometry showed that rat BM‐MSCs at the fifth passage were positive for CD29 (99.0%), CD90 (99.6%), CD44 (92.6%), and negative for CD34 (0.17%) and CD45 (0.24%) (Figure S1). Six weeks after induction of diabetes, diabetic rats exhibited bodyweight loss accompanied by dyslipidemia as evidenced by TG, TC and LDL‐C, but no apparent change was observed in HDL‐C (Figure S2A‐F). MSCs‐treated DN rats exhibited a marked increase in bodyweight but no significant reduction in blood glucose levels. Moreover, MSCs treatment also improved the TG in DN rats, but showed no significant effect on TC, LDL‐C and HDL‐C (Figure S2A‐F).

We also found that protein excretion, blood urea and u‐ACR were increased in DN rats. (Figure 1B,C and D). And these elevated parameters were significantly reduced in DN rats treated with MSCs indicating the improved kidney function (Figure 1B,C and D). Furthermore, HE staining exhibited characteristic tubulointerstitial inflammation including inflammatory cells infiltration and interstitial expansion in kidney section of DN rats. MSCs treatment markedly reduced the inflammatory changes in DN rats (Figure 1E). PAS staining indicated serious histopathological changes, such as diffuse tubular dilation, tubular cell vacuolization, atrophy and detachment in kidney section of DN rats (Figure 1E and F). Following MSCs treatment, tubular injury in DN rats was markedly ameliorated (Figure 1E and F). These results indicated that MSCs could improve histological injury and kidney dysfunction in DN rats.

### 3.2 MSCs transplantation reduces renal inflammation and fibrosis in DN rats

**Figure 2:**
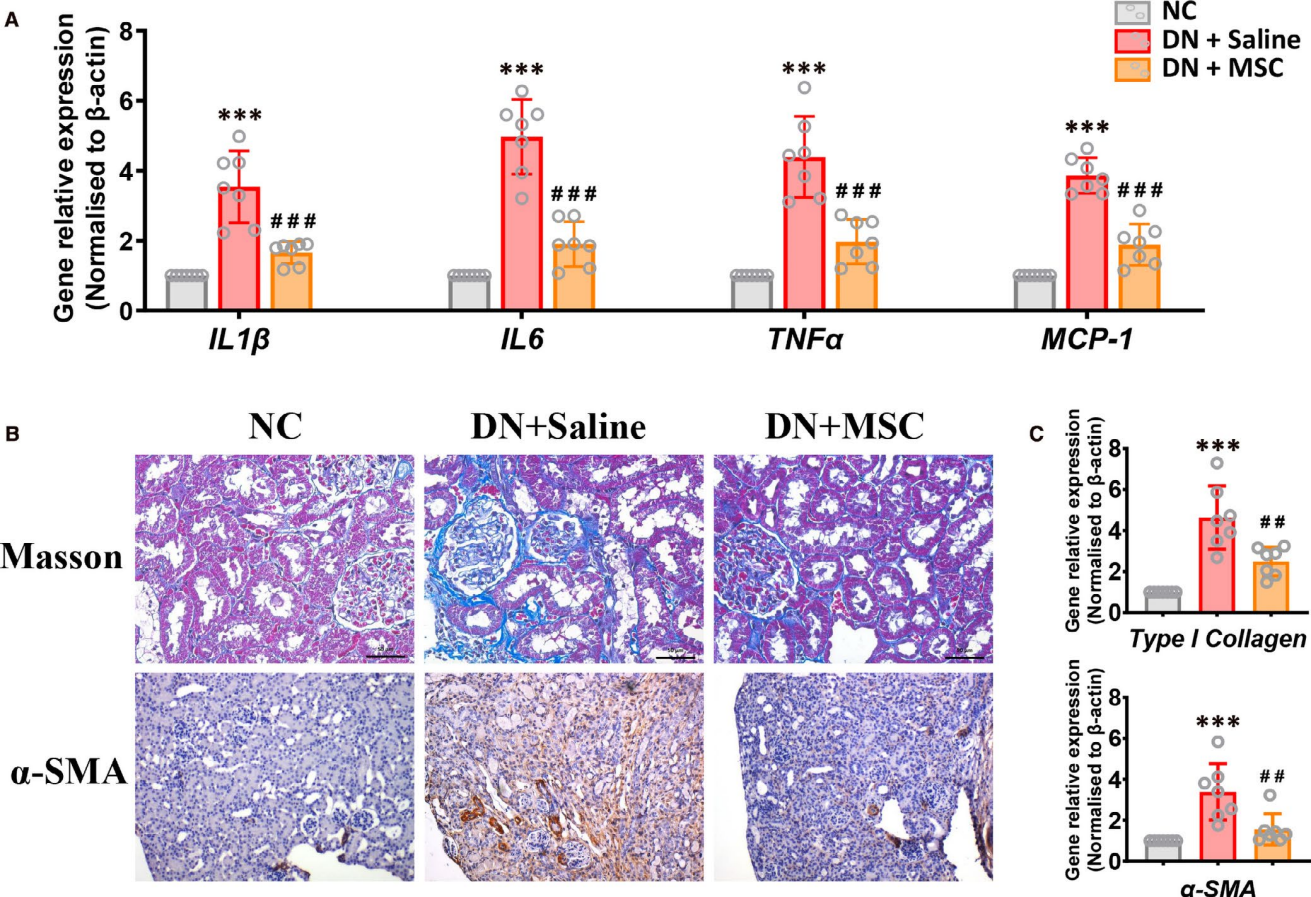
MSCs transplantation attenuates inflammation and fibrosis in DN rats. A, Real‐time PCR analysis of mRNA expression of IL1β, IL6, TNFα and MCP‐1 in kidneys of NC, DN + Saline and DN + MSC after 12 weeks. Data shown are the mean ± SD (n = 7 per group). ***P < .001 vs NC group, ###*P* < .001 vs DN + Saline group. B, Representative Masson's trichrome‐stained (Bar = 50 µm) kidney section and immunohistological staining (Original magnification, ×100) for α‐SMA (marker associated with fibrosis) in kidney section of the normal and DN rats treated with Saline or MSCs after 12 weeks. C, Real‐time PCR analysis of mRNA expression of type I collagen and α‐SMA in kidneys of the normal and DN rats treated with Saline or MSCs after 12 weeks. Data shown are the mean ± SD (n = 7 per group). ***P < .001 vs NC group, ##*P* < .01 vs DN + Saline group

In DN, renal injury is associated with interstitial inflammation and fibrosis. To investigate how MSCs protect against renal injury in DN rats, we tested renal proinflammatory cytokine and chemokine production and investigated the fibrotic kidney lesions of NC, DN + Saline and DN + MSC rats. There is an increased level of proinflammatory cytokine and chemokine production in DN rats. MSCs treatment markedly suppressed mRNA expression of proinflammatory cytokine and chemokines (*IL1β*, *IL6*, *TNF‐a* and *MCP‐1*) (Figure 2A).

Masson staining demonstrated that kidney interstitial fibrosis was improved significantly in MSCs‐treated DN rats compared with DN group rats (Figure 2B). Immunostaining for α‐SMA (marker associated with fibrosis) revealed that the kidneys of DN rats treated with MSCs had less deposition of α‐SMA than DN group rats (Figure 2B). In addition, qPCR showed that *α‐SMA* and *type I collagen* associated with fibrosis was highly expressed in DN group rats, whereas the MSCs‐treated DN rats showed lower expression (Figure 2C). Taken together, these results suggested that MSCs improved renal inflammation and fibrosis in DN rats.

### 3.3 MSCs transplantation selectively reduces CD103^+^ DCs and CD68^+^ CD11c^+^ macrophages in DN rats

**Figure 3:**
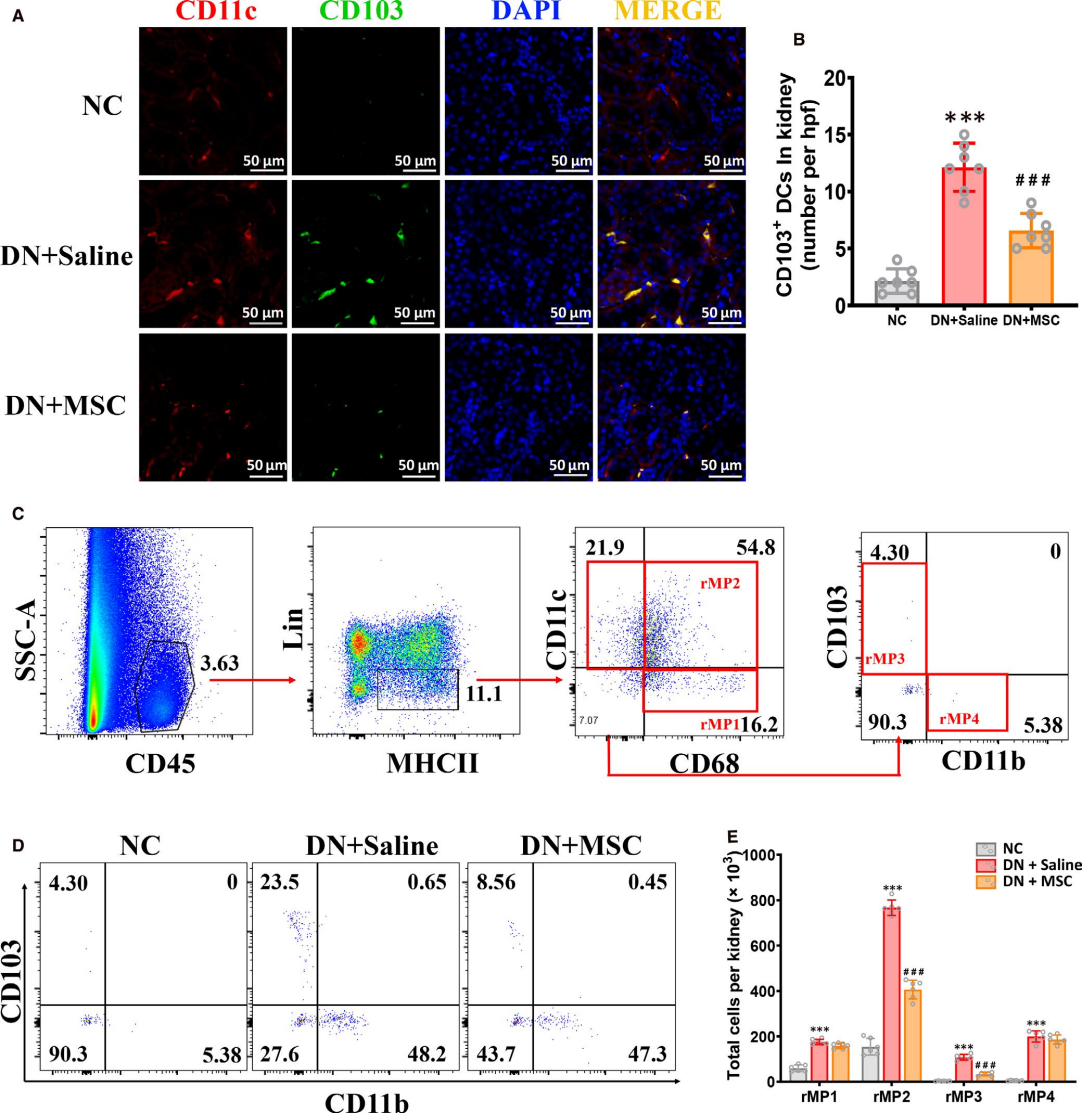
MSCs transplantation depletes renal CD103^+ ^DCs in DN rat. A, Immunofluorescence staining for CD11c (red) and CD103 (green) in kidney section of NC, DN + Saline and DN + MSC rats after 12 weeks. Bar = 50 µm. B, Quantitative analysis of the number of CD11c^+ ^CD103^+ ^DCs in kidneys of NC, DN + Saline and DN + MSC rats. Data shown are the mean ± SD (n = 7 hpf per group, hpf, high‐power field). ***P < .001 vs NC group, ###*P* < .001 vs DN + Saline group. C, Representative FACS analysis showing the gating strategy to identify CD103^+^ DCs in the kidneys. After pregating on CD45^+^ leukocytes, the lin^‐ ^MHC‐II^+^ cells are divided into three populations based on their CD11c and CD68 expression, including CD68^+^ CD11c^‐ ^cells (rMP1), CD68^+ ^CD11c^+^ cells (rMP2) and CD68^‐^ CD11c^+^ cells. CD68^‐^ CD11c^+^ cells are then further divided into CD103^+^ CD11b^‐ ^cells(rMP3) and CD103^−^ CD11b^+^ cells(rMP4). Lin mixture includes CD3, CD19, CD49b, TCR‐β and TCR‐γδ. D, Percentage of CD103^+^ CD11b^‐ ^cells and CD103^‐^ CD11b^+^ cells in kidneys of NC, DN + Saline and DN + MSC rats after 12 weeks. E, Quantitation of the number of four rMP subsets in kidneys of NC, DN + Saline and DN + MSC rats after 12 weeks. Total cell number per kidney is calculated as follows: total cells per kidney × proportion of total cells staining for CD45^+^ subpopulation. Data shown represent the mean ± SD of six rats per group. ***P < .001 vs NC group, ###*P* < .001 vs DN + Saline group

Researches have indicated that renal CD103^+^ DCs are pathogenic in murine CKD.^13^ We then investigated the function of CD11c^+^ CD103^+^ DCs in injured kidney of DN model. IF staining indicated that the CD11c^+^ CD103^+^ cell subsets were significantly increased in DN rats. And MSCs transplantation reduced CD11c^+^ CD103^+^ DCs in kidney of DN rats (Figure 3A and B).

Moreover, a flow cytometry gating strategy was designed to further identify CD103^+^ DCs using kidney tissue from NC, DN + saline and DN + MSC rats. To exclude any contaminating renal epithelial cells, kidney cells were pregated on CD45^+^ leucocytes. Then after gated rMPs as lin^−^ MHCII^+^ cell subsets, rMPs were divided into CD11c^−^ CD68^+^ cells (rMP1), CD11c^+^ CD68^+^ cells (rMP2) and CD11c^+^ CD68^−^ cells. Furthermore, the CD11c^+^ CD68^−^ cells were divided into CD103^+^ DCs (rMP3) and CD11b^+^ DCs (rMP4) (Figure 3C). As shown here, quantification by flow cytometry indicated a highly considerable increase of all four rMPs subsets in kidneys of DN rats compared with normal rats (Figure 3D and E). Notably, we found that DN rats treated with MSCs had a significant decrease in the absolute number and proportion of CD68^+^ CD11c^+^ macrophages and CD103^+^ DCs, but no significant change in CD11b^+^ DCs and CD68^+^ CD11c^–^ macrophages (Figure 3D and E). These data indicate that MSCs selectively reduced kidney CD103^+^ DCs and CD68^+^ CD11c^+^ macrophages in DN rats.

**Figure 4:**
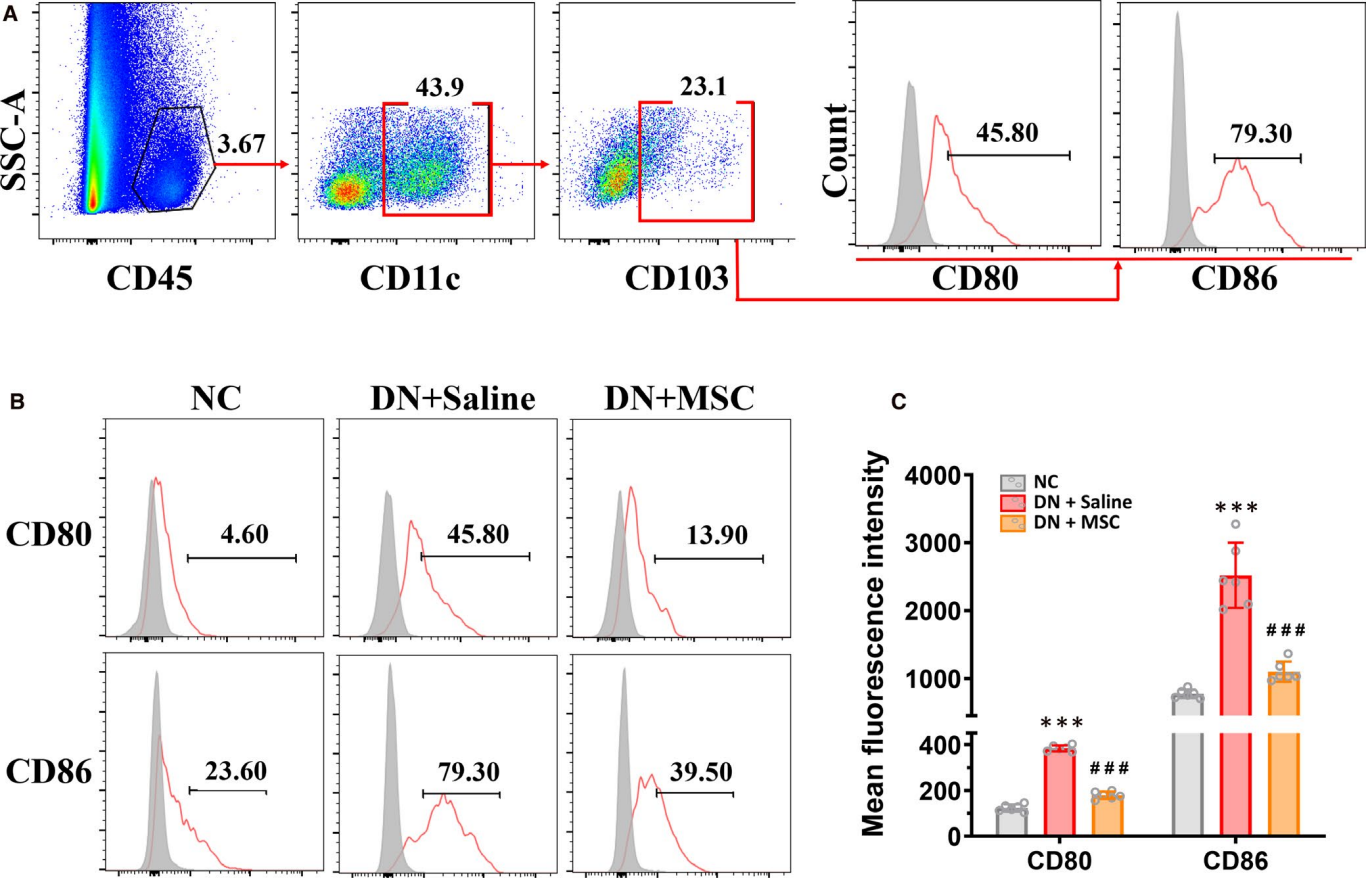
MSCs transplantation suppressed renal CD103^+^ DCs maturation in DN rat. A, Representative FACS analysis showing the gating strategy to identify number and maturation of CD103^+^ DCs in the kidneys. After pregating on CD45^+^ leukocytes, the CD11c^+^ CD103^+^ cells were gated, then the expression of costimulatory factors (CD80, CD86, markers associated with maturation of CD103^+^ DCs) on CD11c^+^ CD103^+^ DCs was analysed. B, Histogram of Representative FACS analysis showing expression of costimulatory factors (CD80, CD86) on CD103^+^ DCs in the kidney of NC, DN + Saline and DN + MSC rats after 12 weeks. Isotype controls (gray‐filled areas) and specific markers (red lines) are shown. C, Flow cytometric analysis of mean fluorescence intensity of costimulatory factors (CD80, CD86) on CD103^+^ DCs in the kidney of NC, DN + Saline and DN + MSC rats after 12 weeks. Data shown are the mean ± SD of evaluations from each group (n = 6 per group). ***P < .001 vs NC group, ###*P* < .001 vs DN + Saline group

Moreover, to exclude any contaminating renal epithelial cells, kidney cells were pregated on CD45^+^ leucocytes, then we gated CD11c^+^ CD103^+^ DCs (Figure 4A). And we found that the CD103^+^ DCs expressed more co‐stimulatory molecules CD80 and CD86 in DN rats compared with normal rats, which suggested that CD103^+^ DCs could up‐regulate cell surface receptors to enhance their ability of activating T cells in vivo (Figure 4B and C). Meanwhile, we found that the CD103^+^ DCs expressed less CD80 and CD86 in kidney of MSCs‐treated DN rats (Figure 4B and C).

These results suggested that MSCs, as specific immunoregulatory factors, could selectively reduce CD103^+^ DCs and CD68^+^ CD11c^+^ macrophages and impact their functions in DN rats.

### 3.4 MSCs transplantation reduces the number and cytotoxic factors of CD8 T cells in DN rats

Studies have also shown that renal CD103^+^ DCs are pathogenic in murine CKD by activating CD8^+^ T cells.^13^ Therefore, we tested the proportion and activation of CD8^+^ T cell in the total renal CD45^+^ leucocytes of DN rats.

**Figure 5:**
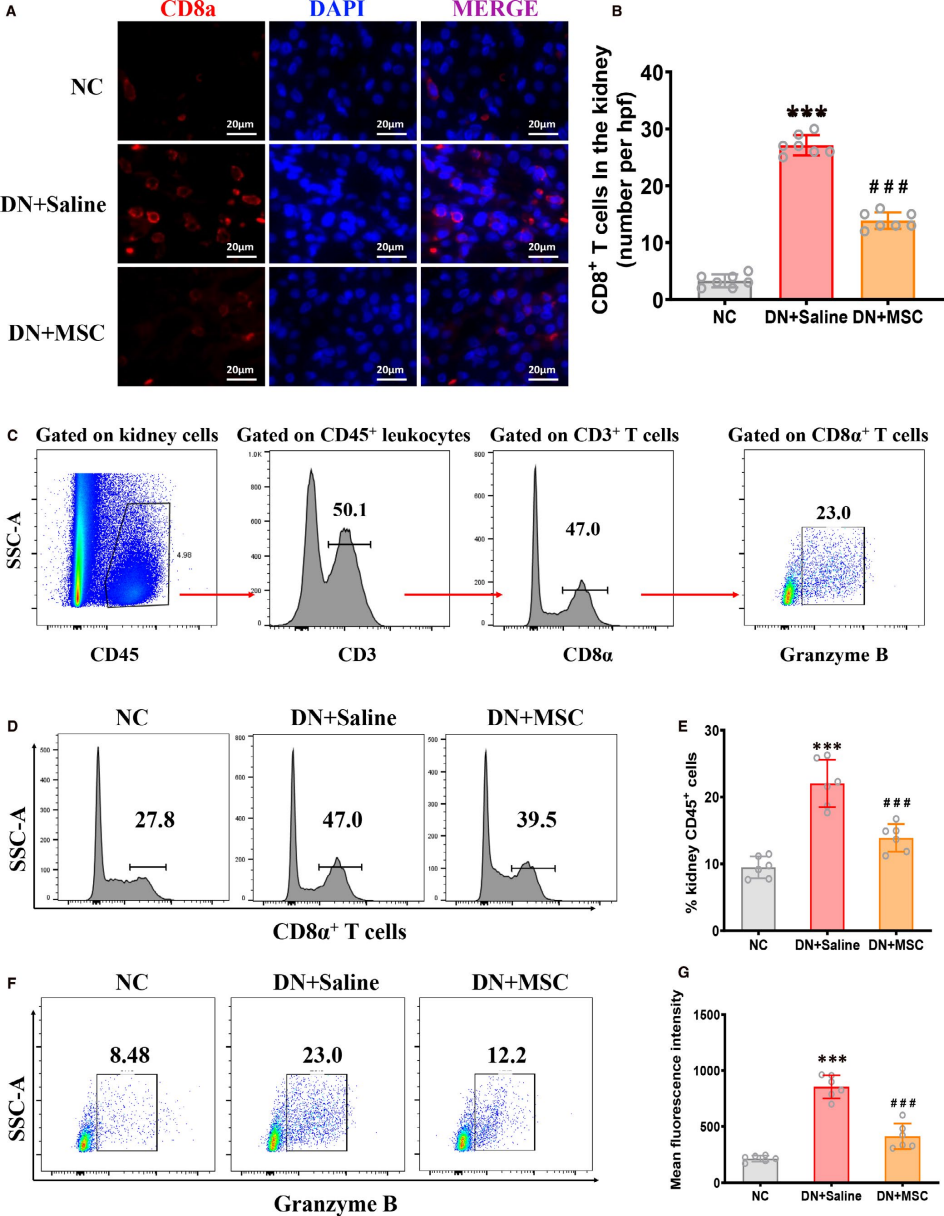
MSCs transplantation reduces CD8 T cells in DN rats. A, Immunofluorescence staining for CD8a (red) and DAPI (blue) in kidney section of NC, DN + Saline and DN + MSC rats after 12 weeks. Bar = 20 µm. B, Quantitative analysis of the number of CD8^+^ T cells in kidneys of NC, DN + Saline and DN + MSC rats after 12 weeks. Data shown are the mean ± SD (n = 7 hpf per group, hpf, high‐power field). ***P < .001 vs NC group, ###*P* < .001 vs DN + Saline group. C, Representative FACS analysis showing the gating strategy to identify CD8^+^ T cells in the kidneys. After pregating on CD45^+^ leukocytes and the CD3^+^ cells, the CD45^+^ CD3^+^ cells are divided into CD8α^+^ and CD8α^‐^ T cells. Then, the expression of Granzyme B (factor associated with activated CD8^+^ T cells) on CD8^+^ T cells was analysed. D, Histogram of representative FACS analysis showing the percentage of CD8α^+^ T cells in the kidneys of NC, DN + Saline and DN + MSC rats after 12 weeks. E, Quantitative analysis of the percentage of CD8^+^ T cells in kidneys of NC, DN + Saline and DN + MSC rats after 12 weeks. Data shown represent the mean ± SD (n = 6 per group). ***P < .001 vs NC group, ###*P* < .001 vs DN + Saline group. F, Representative FACS analysis showing the percentage of the granzyme B expressed in activated CD8α^+^ T cells from kidneys of NC, DN + Saline and DN + MSC rats after 12 weeks. G, Flow cytometric analysis of mean fluorescence intensity of granzyme B expressed in activated CD8α^+^ T cells from kidneys of NC, DN + Saline and DN + MSC rats after 12 weeks. Data shown represent the mean ± SD (n = 6 per group). ***P < .001 vs NC group, ###*P* < .001 vs DN + Saline group

IF staining of renal sections indicated that the CD8^+^ T cells were significantly increased in DN rats compared with normal rats. And MSCs treatment markedly reduced the renal CD8^+^ T cells of DN rats (Figure 5A and B).

Moreover, a flow cytometry gating strategy was designed to further identify CD8^+^ T cells using kidney tissue from NC, DN + saline and DN + MSC rats. Total renal T cells were gated as CD45^+^ CD3^+^ cell subsets and could be divided into two subsets based on CD8α expression. Then after pregating on CD8α^+^ T cells, we investigated the cytotoxic molecules granzyme B in renal CD8 T cells (Figure 5C). Quantification by flow cytometry indicated a highly considerable increase of CD8 T cells in kidneys of DN rats compared with normal rats, and MSCs‐treated DN rats had a markedly decreased proportion of CD8 T cells among renal CD45^+^ leucocytes (Figure 5D and E). Moreover, the renal CD8 T cells expressed more cytotoxic molecule granzyme B in DN rats, and MSCs treatment had markedly reduced the expression of granzyme B in CD8 T cells (Figure 5F and G). These data demonstrated that MSCs might improve renal injury in DN via reducing the number and cytotoxicity of CD8 T cells.

### 3.5 MSCs reduce the population and maturation of CD103^+^ DCs in vitro by down‐regulating cell surface receptors

**Figure 6:**
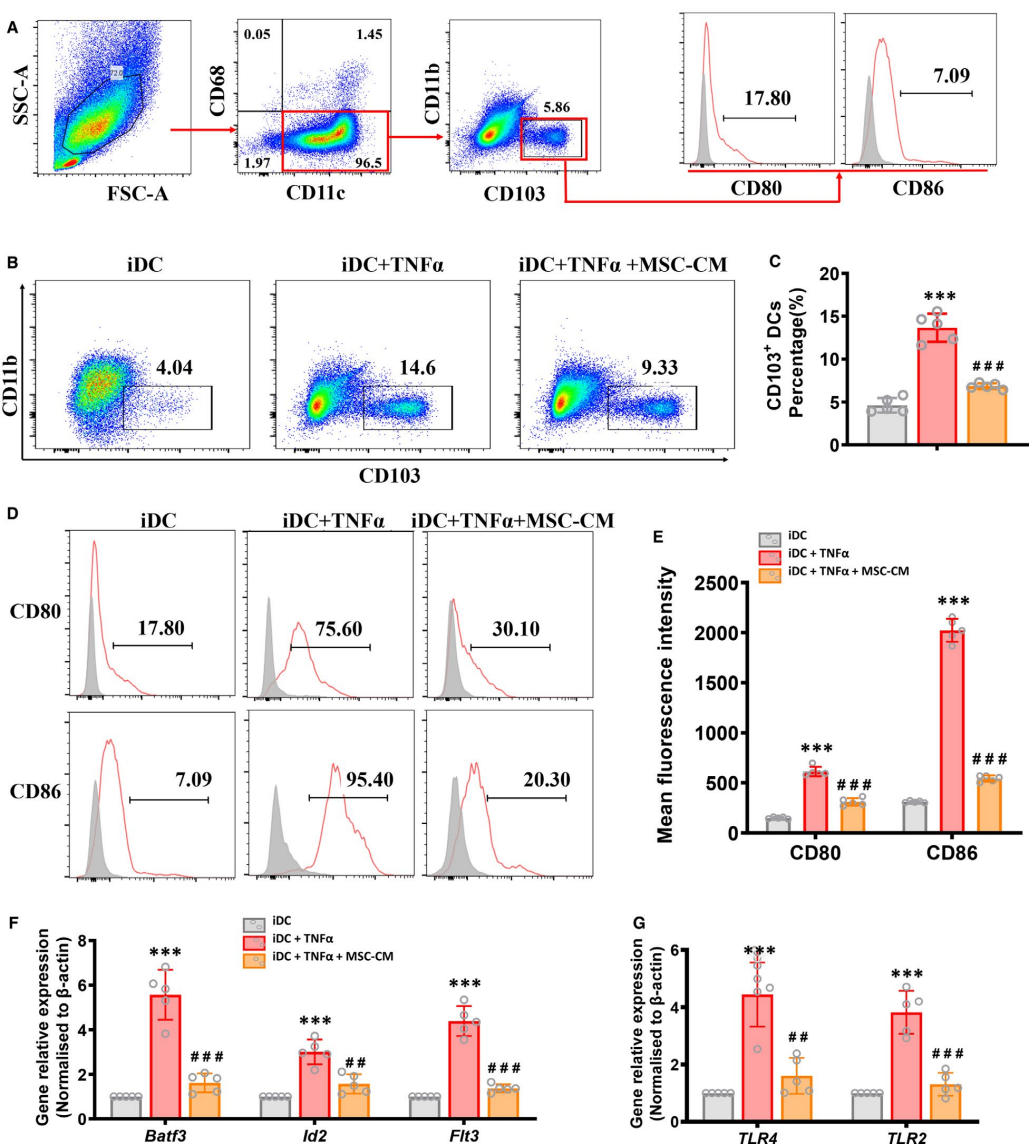
MSCs suppresses DCs maturation in vitro. A, Representative FACS analysis showing the gating strategy to identify CD103^+^ DCs in vitro. Bone marrow‐derived immature DCs were induced with or without TNFα or/and MSC‐CM for 2 days. After pregating on CD68^‐^ CD11c^+^ cells, CD103^+^ CD11b^– ^DCs were gated. Then, the expression of costimulatory factors (CD80, CD86, markers associated with maturation of CD103^+^ DCs) on CD103^+^ CD11b^‐^ DCs was analysed. B and C, Percentage of CD103^+^ CD11b^‐ ^DCs in the group of iDC, iDC + TNFα and iDC + TNFα + MSC‐CM cells. Data shown represent the mean ± SD of each group (n = 5 per group). ***P < .001 vs NC group, ###*P* < .001 vs DN + Saline group. D, Histogram of Representative FACS analysis showing expression of costimulatory factors (CD80, CD86) on CD103^+^ DCs separated from the group of iDC, iDC + TNFα and iDC + TNFα + MSC‐CM cells. Isotype controls (gray‐filled areas) and specific markers (red lines) are shown. E, Flow cytometric analysis of mean fluorescence intensity of costimulatory factors (CD80, CD86) on CD103^+^ DCs separated from the group of iDC, iDC + TNFα and iDC + TNFα + MSC‐CM cells. Data shown are the mean ± SD of each group (n = 5 per group). ***P < .001 vs NC group, ###*P* < .001 vs DN + Saline group. F and G, Real‐time PCR analysis of mRNA expression of transcription factors (Batf3 and Id2), growth factor receptor (Flt3) and Toll‐like receptors (TLR2 and TLR4) in freshly isolated CD103^+^ DCs from the group of iDC, iDC + TNFα and iDC + TNFα + MSC‐CM cells. Data shown are the mean ± SD (n = 5 per group). ***P < .001 vs NC group, ##*P* < .01, ###*P* < .001 vs DN + Saline group

We next tested whether MSCs reduced the population and maturation of CD103^+^ DCs in vitro. A flow cytometry gating strategy was designed to further identify CD103^+^ DCs in the iDCs cultured with or without TNF‐α or/and MSC‐CM. After pregating on CD11c and CD68, we found that more than ninety‐five percent of the cells expressed CD11c and negative of CD68, which indicated that iDCs were obtained successfully via induction with IL4 and GM‐CSF (Figure 6A). We then gated CD103^+^ DCs based on the expression of CD11b and CD103. Moreover, the expression of co‐stimulatory molecules on CD103^+^ DCs was investigated (Figure 6A).

The population of CD11b^−^ CD103^+^ DCs was markedly increased in iDCs induced with TNF‐α compared with those uninduced iDCs. Notably, we found that iDCs cultured with TNF‐α and MSC‐CM for 3 days had a significant reduction in the population of CD103^+^ DCs compared with iDCs only induced with TNF‐α (Figure 6B and C).

In addition, the CD80 and CD86 expression was markedly increased on CD103^+^ DCs among TNF‐α‐induced iDCs compared with uninduced iDCs, which indicated that CD103^+^ DCs could up‐regulate cell surface receptors to enhance their ability of activating T cells in vitro. Interestingly, we found that iDCs treated with TNF‐α and MSC‐CM for 3 days had a markedly reduced expression of CD80 and CD86 on CD103^+^ DCs, suggesting that MSC‐CM could suppress the maturation of CD103^+^ DCs in vitro by down‐regulating cell surface receptors (Figure 6D and E).

We next investigated the selective expression of growth factor receptor and transcription factors on DCs subsets. *Batf3* and *Id2* (important transcription factors for development of CD8α^+^ DCs) were expressed lower on CD103^+^ DCs cultured with MSC‐CM (Figure 6F). Moreover, *Flt3* (growth factor receptor for DCs), which is important for the CD103^+^ DCs development, showed a reduced expression on CD103^+^ DCs cultured with MSC‐CM (Figure 6F). In addition, the Toll‐like receptor *TLR2* and *TLR4* (damage‐associated molecular pattern receptors, DAMPR) exhibited a significantly increased expression on CD103^+^ DCs induced with TNF‐α, with a reduced expression when cultured with MSC‐CM (Figure 6G).

These results suggested that MSCs, as specific immunoregulatory factors, could selectively reduce the proportion of CD103^+^ DCs and impact their functions by suppressing the expression of receptors on CD103^+^ DCs.

### 3.6 MSCs suppress CD103^+^ DC‐mediated CD8 T cell proliferation and activation

**Figure 7:**
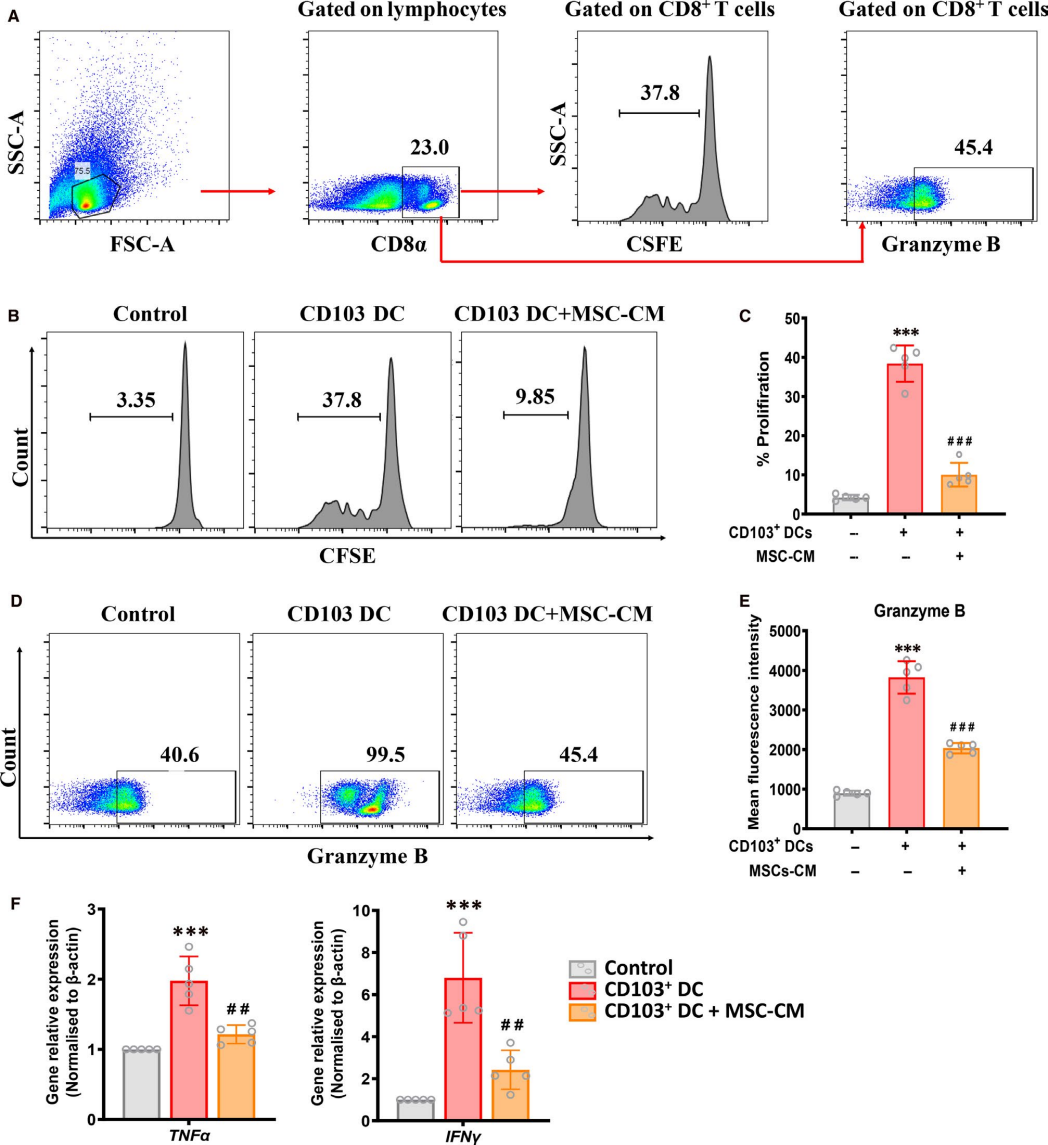
MSCs suppresses CD103^+^ DCs‐mediated CD8 T cells proliferation and activation. A, Representative FACS analysis showing the gating strategy to identify CD8^+^ T cells in vitro. Spleen mononuclear cells were incubated with CFSE and cocultured with or without CD103^+^ DCs (sorted form TNFα‐induced DCs) or/and MSC‐CM for 2 days. Then after pregating on CD8α^+^ T cells, CFSE intensity and the expression of Granzyme B in CD8α^+^ T cells was analysed. B and C, Percentage of CD8α^+^ T cells in the group of CD8α^+^ T cells cocultured with or without CD103^+^ DCs (sorted form TNFα‐induced DCs) or/and MSC‐CM for 2 days. Data shown represent the mean ± SD of each group (n = 5 per group). ***P < .001 vs NC group, ###*P* < .001 vs DN + Saline group. D, Representative FACS analysis showing expression of Granzyme B on CD8α^+^ T cells in the group of CD8α^+^ T cells cocultured with or without CD103^+^ DCs (sorted form TNFα‐induced DCs) or/and MSC‐CM for 2 days. E, Flow cytometric analysis of mean fluorescence intensity of Granzyme B on CD8α^+^ T cells in the group of CD8α^+^ T cells cocultured with or without CD103^+^ DCs (sorted form TNFα‐induced DCs) or/and MSC‐CM for 2 days. Data shown are the mean ± SD of each group (n = 5 per group). ***P < .001 vs NC group, ###*P* < .001 vs DN + Saline group. F, Real‐time PCR analysis of mRNA expression of TNFα and IFNγ in freshly isolated CD8α^+^ T from the group of CD8α^+^ T cells cocultured with or without CD103+ DCs (sorted form TNFα‐induced DCs) or/and MSC‐CM for 2 days. Data shown are the mean ± SD (n = 5 per group). ***P < .001 vs NC group, ##*P* < .01 vs DN + Saline group

Next, we examined whether MSCs impacted the proliferation and activation of CD8 T cells by suppressing CD103^+^ DCs. First, we assessed the role of MSCs on CD8 T cell priming capability mediated by CD103^+^ DCs in vitro. Spleen mononuclear cells were pregated on CD8α^+^ T cells, then CFSE intensity and the Granzyme B expression in CD8α^+^ T cells was analysed (Figure 7A). CD103^+^ DCs, separated from TNF‐α induced mDCs, showed higher priming capacity than control CD103^+^ DCs in the CD8 T cell proliferation assay; however, CD103^+^ DCs, separated from TNF‐α induced mDCs pre‐treated with MSC‐CM, showed significantly lower CD8 T cell priming capacity (Figure 7B and C). In addition, when cocultured with CD103^+^ DCs separated from TNF‐α induced mDC, CD8 T cells exhibited high expression of granzyme B, as well as mRNA levels of *TNF‐α* and *IFNγ*, which indicated that CD103^+^ DCs, separated from TNF‐α induced mDC, had increased ability of activating CD8 T cells. However, MSC‐CM pre‐treated CD103^+^ DCs had decreased ability of activating CD8 T cells (Figure 7D,E and F).

Moreover, we have shown that MSCs suppressed the maturation of CD103^+^ DCs (Figure 6D and E), which might partially explain how MSCs impact the proliferation and activation of CD8 T cells by suppressing CD103^+^ DCs.

## 4 DISCUSSION

**Figure 8:**
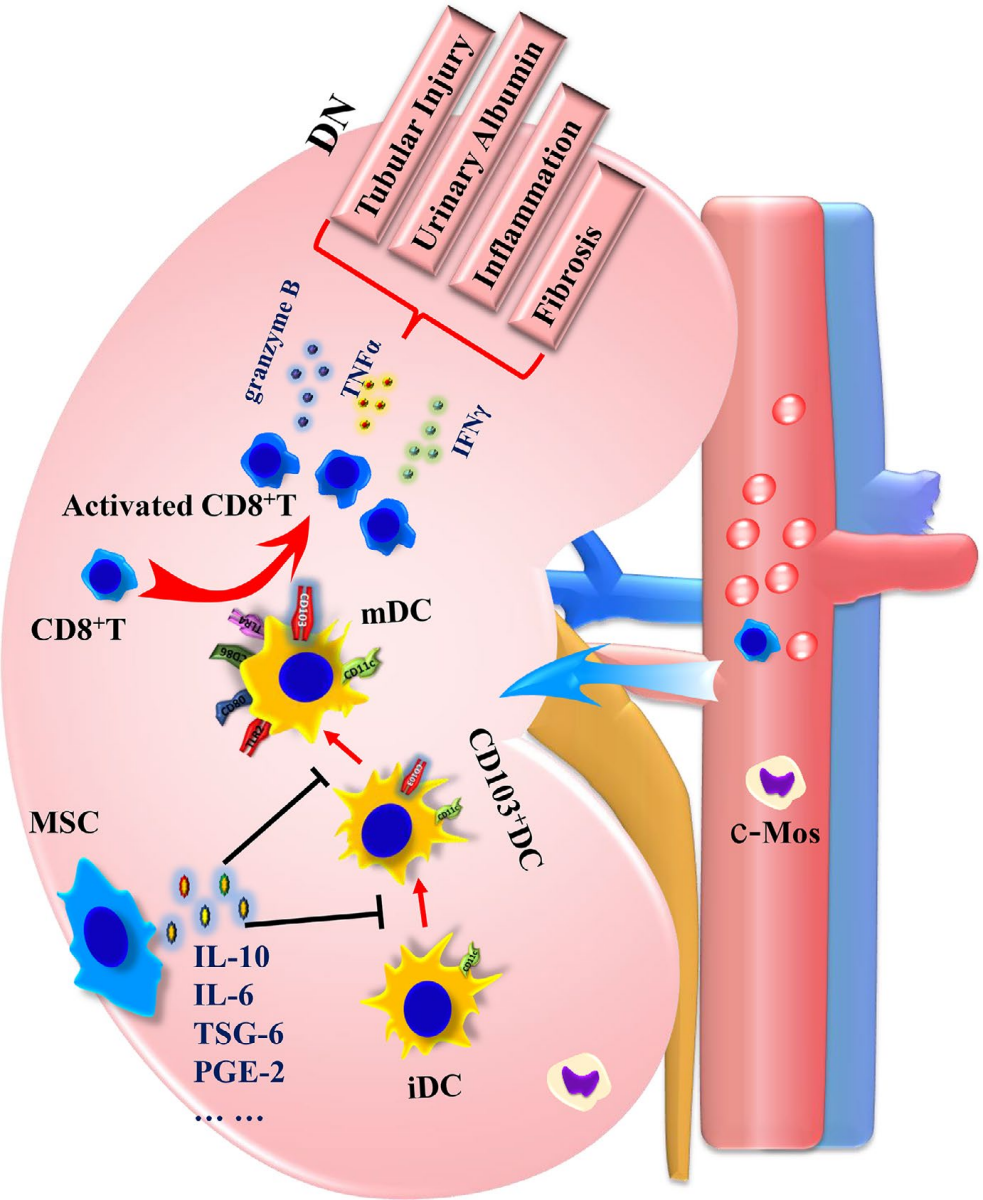
A proposed cellular and molecular model of the mechanisms underlying MSCs transplantation on diabetic nephropathy (DN). The consistent high blood glucose may lead inflammation and injury to the kidney, causing the accumulation and maturation of CD103^+^ DCs. Then, CD8^+ ^T cells are activated by the mDCs. The activated CD8^+^ T cells proliferated rapidly and release Granzyme B, TNFα and IFNγ, causing disfunction, structural injury, and local inflammation and fibrosis of the kidney more serious in DN rats. MSCs transplantation indirectly impacted the proliferation and activation of CD8 T cell via reducing the number and suppressing the maturation of CD103^+^ DCs, thus protecting the kidney. (c‐Mos, circulating monocytes; iDCs, immature dendritic cells; mDCs, mature dendritic cells)

In this study, we have again confirmed the immunosuppressive effect of MSCs in the kidney of DN rat. First, we found that MSCs transplantation notably improved kidney disfunction and structural injury, and ameliorated kidney local inflammation and fibrosis in DN rats. Then, we found that both CD103^+^ DCs and CD8^+^ T cells were notably decreased in MSCs‐treated DN rats. Meanwhile, the maturation of CD103^+^ DCs was significantly suppressed as shown by reduced expression of CD80 and CD86. In addition, we have detected that the cytotoxic factors of CD8^+^ T cells were markedly increased in DN rats, but decreased in MSCs‐treated DN rats. Moreover MSC‐CM indirectly impacted the proliferation and activation of CD8 T cell via reducing the number and suppressing the maturation of CD103^+^ DCs in vitro. The kidney protective effect of MSCs may be partly related to their suppression effect on renal CD103^+^ DC‐mediated CD8^+^ T cell responses in DN rats, through secreting multiple soluble factors^19^, ^21^, ^29^ (Figure 8). This study demonstrated MSCs as a promising cell resource to cure DN.

Allogeneic MSCs have prolonged skin graft survival in baboons,^30^ shown to be as effective as syngeneic MSCs for Therapeutic Revascularization^31^ and in enhancing wound healing in mice,^32^ and prolonged graft survival,^33^ yet failed to prolong survival of heterotopic heart transplants in rats, showed accelerated rejection in some studies.^34^, ^35^ The exact nature of the immune response to allogeneic MSCs still remains poorly characterized. However, considering better ensure the effect of MSCs intervention to explore the mechanism of renal protection, we still used the syngenic rat‐derived MSCs in this study.

Mesenchymal stem cells can maintain the homeostasis of immune microenvironment of the tissues.^7^, ^12^, ^13^ The homing of MSCs is based on the changes of local tissue injury or inflammation.^14^, ^15^ Considering that the renal tissue of healthy rats is in a good steady state, we did not include the group of MSCs‐treated healthy rats in this study. Of course, we feel that if healthy rats receive MSCs intervention, there may be a good effect to a certain extent. More studies are needed to reveal the effect of MSCs on healthy rats. DCs are important for the initiation of T cell responses through up‐regulating a few immunomodulating factors including co‐stimulatory, integrin and adhesion molecules.^36^ Studies have reported CD103^+^ DCs as an unique subset of DCs in diverse non‐lymphoid organs, such as the intestine, kidney, dermis and lung.^13^, ^37^, ^38^, ^39^ It has been demonstrated that kidney CD103^+^ DCs were pathogenic via mediating CD8^+^ T cells responses in AN^13^ and that Flt3 inhibitor AC220 effectively suppressed CD103^+^ dendritic cell and reduced kidney injury in AN mouse.^18^ While study has also found that the adoptive transfer of CD103^+^ DCs to Batf3 deficient mice with steatohepatitis significantly alleviated the steatosis with improvement of inflammation and cell injury.^40^ Another study has demonstrated that the administration of Flt3 ligand alleviated chronic ileitis via increasing the CD103^+^ DCs.^37^ A proposed hypothesis on these distinct roles of CD103^+^ DCs could be associated with the unique microenvironments they exposure in different local, stages or types of disease.^18^ Consistently high blood glucose may lead to complicated inflammation microenvironments in different stages of DN. So, the CD103^+^ DCs might also play an important role in the progression of renal injury in DN, and CD103^+^ DCs might be the potential target for DN treatment. MSCs were widely used to treat DN in the last decade for their production of multiple factors.

Extensive studies have shown that MSCs could reduce expression of CD80 and CD86 on DCs to attenuate their ability of antigen presentation through secreting tumour necrosis factor‐inducible gene 6 protein (TSG6), IL6 and prostaglandin E2 (PGE2).^19^, ^21^, ^41^ It has been recently reported that MSCs suppressed DCs maturation via regulating the phosphorylation of Stat1 and Stat6 in experimental autoimmune uveitis.^20^ Consistent with these observations, we have confirmed that the renal CD103^+^ DCs also exhibited a pathogenic effect in DN rat. In addition, MSCs are not only able to reduce the number of CD103^+^ DCs, but also show consistent suppressive effect on CD103^+^ DCs both in vitro and in vivo. Previous research has demonstrated that kidney CD103^+^ DCs exhibited significantly higher expression of *Batf3* and *Id2*, *Flt3* and DAMP receptors (*TLR2* and *TLR4*) in AN mice compared with normal mice.^13^ Here, we found that CD103^+^ DCs also showed increased mRNA expression of *Batf3*, *Id2*, *Flt3*, *TLR2* and *TLR4*, which may play a vital role in the function and development of CD103^+^ DCs. Thus, the suppressive effect of MSCs on CD103^+^ DCs may be partly associated with the decreased expression of *Batf3*, *Id2*, *Flt3*, *TLR2* and *TLR4*. For example, MSCs might derive multiple soluble factors, which may interact with the transcription factors, growth factor and DAMP receptors on CD103^+^ DCs to exhibit immunosuppressive effect. However, more experiments are required to reveal the underlying suppressive mechanism of MSCs on CD103^+^ DCs. Importantly, MSCs treatment could regulate the homeostasis of CD103^+^ DCs, which might be helpful to understand the potential protective mechanisms of MSCs in DN. Meanwhile, we also observed that MSCs treatment caused an obvious reduction of CD11c^+^ CD68^+^ macrophages, but not CD11b^+^ DCs and CD11c^−^ CD68^+^ cells. So, it is possible that the protective effect of MSCs could partially relate to their targeting on other immune cells besides CD103^+^ DCs. Here, we focus on the role of renal CD103^+^ DCs in DN rat with or without MSCs treatment. However, more experiments are required to identify the function of renal CD11c^+^ CD68^+^ macrophages in DN rat.

Study has shown that CD103^+^ DCs tend to enhance the responses of CD8 T cells during respiratory influenza infection in lung.^42^ Another study demonstrated the liver CD103^+^ DCs serve as an important antigen‐presenting cells (APCs) to support CD8 T cell responses in respond to hepatotoxic viral infections.^43^ Furthermore, it is reported that renal CD103^+^ DCs could capture antigens and present them to CD8 T cells in immunity‐mediated nephritis.^13^ Consistent with these studies, our results revealed that CD103^+^ DCs either in the kidney of DN rat or induced by TNF‐α in vitro showed enhanced capacity of priming CD8 T cell responses; however, MSC‐CM pre‐treated CD103^+^ DCs showed lower CD8 T cell priming capacity. Other possible causes that might mediate the improvement of renal injury in DN rat treated with MSCs may be related to the declined levels of *IL1β*, *IL6*, *TNF‐α* and *MCP‐1*, which played an important role in the activation and infiltration of inflammatory cells in diseased kidney. And the fibrosis amelioration of MSCs treatment might be partly associated with the reduced local inflammation in the kidney.

## 5 CONCLUSION

In conclusion, MSCs can significantly alleviate renal injury in DN, with distinct improvement of kidney function, inflammation and fibrosis. Our data exhibit important evidence for MSCs targeting the pathogenic CD103^+^ DCs to treat DN. These results indicate a possible underlying mechanism for MSCs to improve DN through immunosuppressive ways.

## CONFLICT OF INTEREST

The authors declared that there are no conflicts of interest.

## AUTHOR CONTRIBUTIONS

FPZ and CSW designed the experiment and interpreted results. FPZ drafted manuscript. FPZ, YC, XW, RWM, DLC and LL performed experiments. JPL, YNC, JQC and YRL made critical revision to manuscript. All authors have read and approved the final manuscript. FPZ and CSW contributed equally to this work.

## Supporting information

Fig S1‐S2

Table S1

## Data Availability

The data that support the findings of this study could be obtained upon reasonable request to the corresponding author.
