# Supplementary material for: Mesenchymal stem cells alleviate rat diabetic nephropathy by suppressing CD103+ DCs‐mediated CD8+ T cell responses
Source: J Cell Mol Med. 2020 Apr 13;24(10):5817–31. doi: 10.1111/jcmm.15250 (PMC7214166; doi:10.1111/jcmm.15250)
Supplement: Supplementary file 1 — Fig S1‐S2 [file JCMM-24-5817-s001.docx]

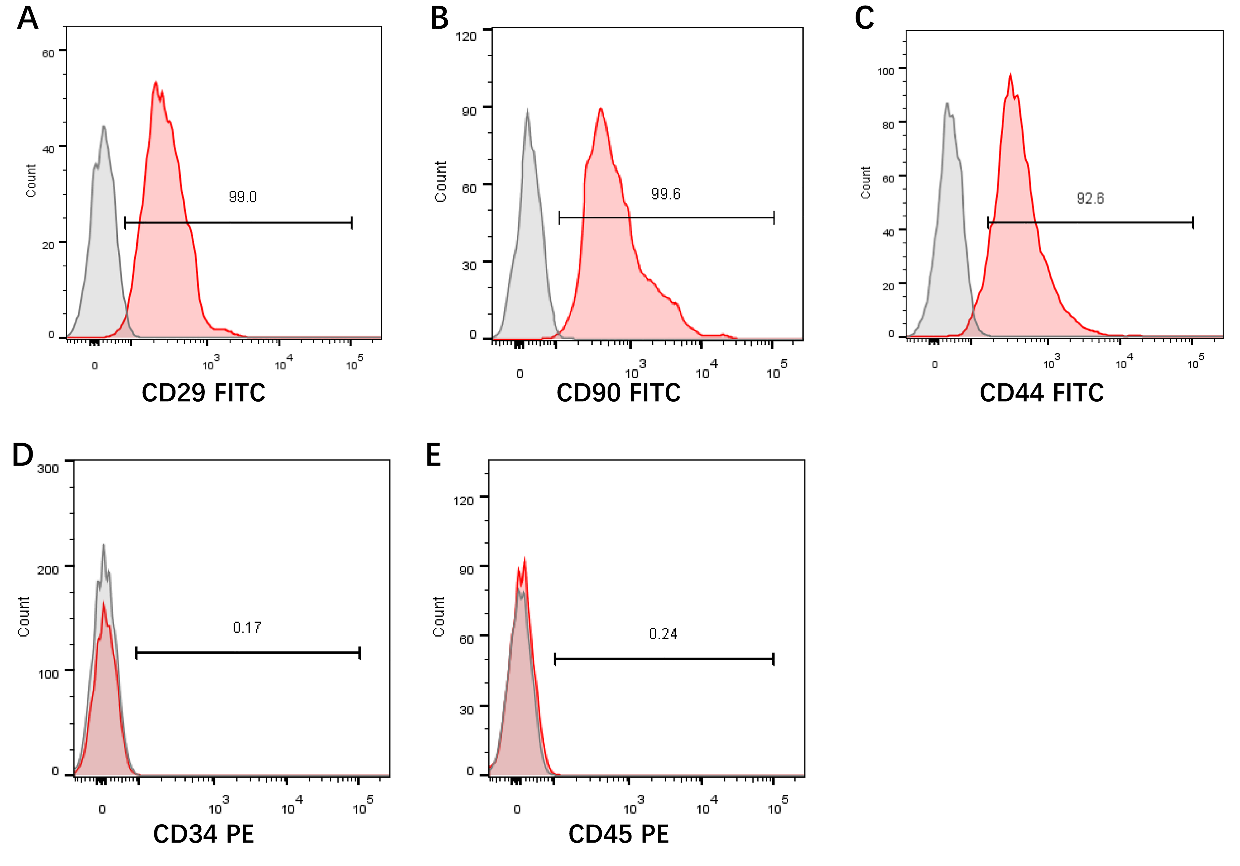


**Figure S1.** **Phenotype of rat BM-MSCs.**

Flow cytometry analysis of immune-markers in rat BM-MSCs. Results confirmed that rat BM-MSCs were positive for CD29 (A), CD90 (B) and CD44 (C), (B) but negative for CD34 (D) and CD45(E).


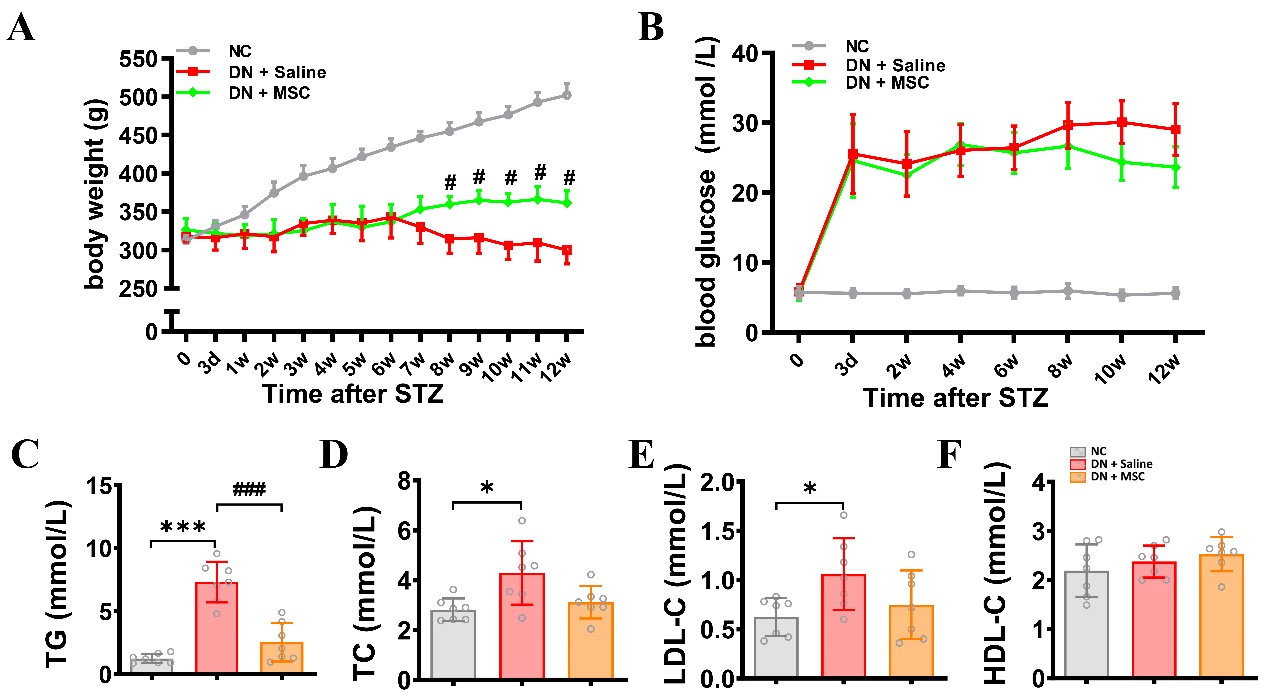


**Figure S2. Effects of MSCs on blood glucose, body weight, and** **lipid profiles in DN rats.**

(A) body weight, (B) Blood glucose level, (C) triglycerides (TG), (D) cholesterol (TC), (E) low-density lipoprotein (LDL-C), (F) high-density lipoprotein (HDL-C) in NC, DN + saline and DN + MSC. Data are shown as the means ± SD (n=7). (**P <* 0.05, ****P <* 0.001 *vs* NC, ^#^*P* < 0.05 *vs* DN).
