# Supplementary material for: Mesenchymal stem cells alleviate rat diabetic nephropathy by suppressing CD103+ DCs‐mediated CD8+ T cell responses
Source: J Cell Mol Med. 2020 Apr 13;24(10):5817–31. doi: 10.1111/jcmm.15250 (PMC7214166; doi:10.1111/jcmm.15250)
Supplement: Supplementary file 2 — Table S1 [file JCMM-24-5817-s002.docx]

Table S1. Primer sequences used for quantitative PCR.

| Target | Species | Primer sequences (5'→3') |
| --- | --- | --- |
| *IL-1β* | Rat | F: ATCTCACAGCAGCATCTCGACAAG  R: CACACTAGCAGGTCGTCATCATCC |
| *IL-6* | Rat | F: AGGAGTGGCTAAGGACCAAGACC  R: TGCCGAGTAGACCTCATAGTGACC |
| *TNF-α* | Rat | F: ATGGGCTCCCTCTCATCAGTTCC  R: GCTCCTCCGCTTGGTGGTTTG |
| *MCP-1* | Rat | F: GCAGGTCTCTGTCACGCTTCTG  R: GAATGAGTAGCAGCAGGTGAGTGG |
| *Collagen type I* | Rat | F: TGTTGGTCCTGCTGGCAAGAATG  R: GTCACCTTGTTCGCCTGTCTCAC |
| *α-SMA* | Rat | F: GCCACTGCTGCTTCCTCTTCTTC  R: CCCGCCGACTCCATTCCAATG |
| *Batf3* | Rat | F: AGTCTGGAGCAGGAGAACTCTGTG  R: TCCTTCAGCGCCTCAGTCAGG |
| *Id2* | Rat | F: AGCCTGCACCACCAGAGACC  R: AATTCAGACGCCTGCAAGGACAG |
| *Flt3* | Rat | F: AGAGGCTGGAAGAAGAGGAGGAAG  R: GCTGCCAGGTCTCTGTGAACAC |
| *TLR2* | Rat | F: TCTGGAGTCTGCTGTGCCCTTC  R: GGAGCCACGCCCACATCATTC |
| *TLR4* | Rat | F: TTGCTGCCAACATCATCCAGGAAG  R: CAGAGCGGCTACTCAGAAACTGC |
| *IFN-γ* | Rat | F: AAGACAACCAGGCCATCAGCAAC  R: GAACTTGGCGATGCTCATGAATGC |
| *β-actin* | Rat | F: GCAAATGCTTCTAGGCGGAC  R: AAGAAAGGGTGTAAAACGCAGC |
